# Combination of spectral index and transfer learning strategy for glyphosate-resistant cultivar identification

**DOI:** 10.3389/fpls.2022.973745

**Published:** 2022-08-08

**Authors:** Mingzhu Tao, Yong He, Xiulin Bai, Xiaoyun Chen, Yuzhen Wei, Cheng Peng, Xuping Feng

**Affiliations:** ^1^College of Biosystems Engineering and Food Science, Zhejiang University, Hangzhou, China; ^2^Key Laboratory of Traceability for Agricultural Genetically Modified Organisms, Ministry of Agriculture and Rural Affairs, Zhejiang Academy of Agricultural Sciences, Hangzhou, China; ^3^School of Information Engineering, Huzhou University, Huzhou, China

**Keywords:** decision model, glyphosate resistance, hyperspectral imaging, source domain updating, support vector machine, transfer component analysis

## Abstract

Glyphosate is one of the most widely used non-selective herbicides, and the creation of glyphosate-resistant cultivars solves the problem of limited spraying area. Therefore, it is of great significance to quickly identify resistant cultivars without destruction during the development of superior cultivars. This work took maize seedlings as the experimental object, and the spectral indices of leaves were calculated to construct a model with good robustness that could be used in different experiments. Compared with no transfer strategies, transferability of support vector machine learning model was improved by randomly selecting 14% of source domain from target domain to train and applying transfer component analysis algorithm, the accuracy on target domain reached 83% (increased by 71%), recall increased from 10 to 100%, and F1-score increased from 0.17 to 0.86. The overall results showed that both transfer component analysis algorithm and updating source domain could improve the transferability of model among experiments, and these two transfer strategies could complement each other’s advantages to achieve the best classification performance. Therefore, this work is beneficial to timely understanding of the physiological status of plants, identifying glyphosate resistant cultivars, and ultimately provides theoretical basis and technical support for new cultivar creation and high-throughput selection.

## Introduction

High efficiency and low cost make herbicides become an important means in weed management (Pan et al., 2019). Among them, glyphosate is considered as one of the best herbicides with superior quality, excellent performance, low toxicity and broad grass removal spectrum (Duke and Powles, 2008). Glyphosate acts on shikimic acid pathway in plants (Gomes et al., 2014) and inhibits the synthesis of aromatic amino acid and compounds related to protection mechanisms (Corrêa et al., 2016), thereby adversely affecting plants physiology (Van Bruggen et al., 2018). Once glyphosate comes into contact with green plants (whether weeds or crops), it can be absorbed by stems, leaves and other organs. The physiological balance and internal structure of the plant can be destroyed by glyphosate and finally causes wither and die (Van Bruggen et al., 2018; Lin et al., 2023). Therefore, the non-selectivity of glyphosate drives breeders to create resistant cultivars to break the limit for glyphosate use (Clapp, 2021). It can be sprayed after harvest and even throughout the crop growth cycle to ensure crop yield while reducing the labor cost of weed management in the field.

Generally, many *in vitro* culture and field screening verifications are often required in the process of new glyphosate resistant cultivars creation. Common screening methods including visual observation and bioassays, take 10–14 days from spraying glyphosate to resistant identification (Singh et al., 2021), which is time-consuming and labor-intensive. Hence, exploring a rapid non-destructive detection of glyphosate-tolerant cultivar method can speed up the breeding process.

Hyperspectral imaging (HSI) technology can obtain the images and spectra of samples simultaneously (Zea et al., 2022). Images of different bands reflect the external shape and texture from multiple angles. Spectra reveals the differences of chemical substances in samples through the reflectance value in different bands (Shirzadifar et al., 2020a; Zhang et al., 2021b). As the derived parameter of spectral reflectance, the spectral index is composed of multiple band combination by linear or non-linear methods, and has more abundant information compared with multiple single bands. Besides, multivariate data analysis can help uncover useful information hidden within it (Maione et al., 2019), especially for massive datasets from sensors. Machine learning methods showed the excellent data mining ability in hyperspectral data mining (Yang et al., 2020; Najafabadi, 2021; Weng et al., 2021), and the combination between them can be exploited as a competent tool in plant science (Greener et al., 2022) such as early stress detection (Gu et al., 2019; Lu et al., 2020; Zheng et al., 2020), unsound kernel identification (Liang et al., 2020; Zhang et al., 2021a), and the evaluation of nutrition content (Zhang et al., 2020a,b; Najafabadi, 2021).

Generally speaking, an optimal machine learning model can achieve satisfactory results based on specific data sets (An et al., 2022; Greener et al., 2022). But it may not match the features of other data sets with the same type. The property of spectral data was influenced by plant grown, experiment design, instrument status (Qiu et al., 2020), which greatly limits the robustness and generalization of the model. On the other hand, excellent machine learning model depends on adequate data (Zhu et al., 2020; Greener et al., 2022), while it is time-consuming to obtain a sufficient number of samples of the new condition. To resolve this problem, transfer learning has been introduced. By transferring historical knowledge to new task (Cheplygina et al., 2019; Talo et al., 2019), it exhibits great potential in dealing with the situation where training set and test set come from different data distribution including hyperspectral data (Tao et al., 2019; Zhu et al., 2020). Previous literature (Tao et al., 2019) reported a transferable spectroscopic diagnosis model to predict soil arsenic concentration in other areas, not limited to a specific area. Accordingly, it is viable to apply transfer strategies to solve the heterogeneity of samples of different experiments.

Therefore, HSI technology is a powerful tool to rapidly screen target cultivars and accelerate the breeding process. The spectral index can be used to evaluate the state of plant growth. Machine learning can fully mine spectral information to improve model performance on the test set. And emerging transfer learning can further improve model performance in terms of universality on various datasets. However, there are few researches on the detection of glyphosate-tolerant cultivars based on spectral indices of leaves of maize seedling, not to mention the transferability of machine learning model between different experiments.

In this study, we aimed to propose a high-throughput rapid non-destructive model for identifying glyphosate-resistant cultivars which could be used to screen new samples from different times. Specifically, the following questions were discussed: (1) what was the difference in spectral index between glyphosate resistant and sensitive cultivars? (2) how to build a robustness model for identifying glyphosate resistant cultivar? (3) could the transfer strategy improve the classification model? By responding to the above questions, this research could help breeders timely understand the physiological conditions of plant stress, complete the detection of glyphosate-tolerant cultivars, and ultimately provide theoretical basis and technical support for new cultivar creation.

## Materials and methods

### Sample preparation

Two maize cultivars, glyphosate-resistant and glyphosate-sensitive, were designated as R and S, respectively. Glyphosate resistance of R maize was obtained by expression of mutant 5-enolpyruvylshikimate-3-phosphate synthase enzyme. All the seeds were provided by the Institute of Insect Science, Zhejiang University, Hangzhou, China. The detailed information on these two seeds was introduced in our previous study (Feng et al., 2018). Three independent experiments were conducted in August, November, and December 2021, designated as Exp.1, Exp.2, and Exp.3, respectively. In each experiment, these maize plants were grown in the same artificial climate chamber. The temperature and photoperiod of day/night were 28/26°C and 11/13 h, respectively. The average relative humidity was adjusted to 55%. Treatment group and control group were set up for both cultivars. In order to avoid the possible effects of the glyphosate from treatment group on the control group, these two groups were placed in two identical artificial climate chambers respectively. When maize plants grew to the three-leaf stage (the third leaf was fully expanded but the fourth leaf was not), the maize plants of treatment group were subjected to glyphosate, while the maize plants of control group were sprayed with water.

### Visible/near-infrared hyperspectral image acquisition

**FIGURE 1:**
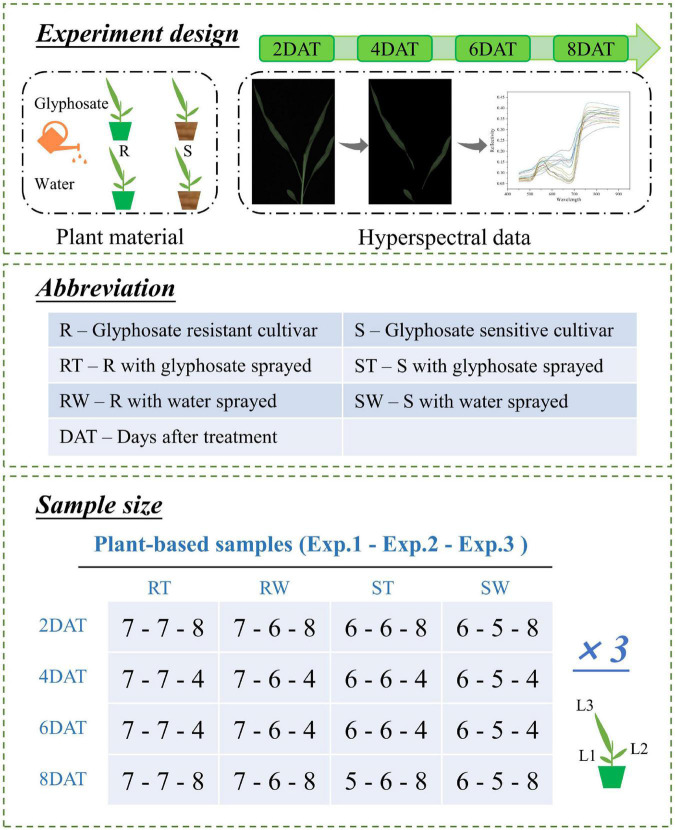
Schematic diagram and detailed information for experiments. Three independent experiments were conducted in August, November, and December 2021, designated as Exp.1, Exp.2, and Exp.3, respectively. In each experiment, treatment group and control group were set up for both cultivars (glyphosate resistant and glyphosate sensitive cultivar). When maize plants grew to the three-leaf stage, the maize plants of treatment group were subjected to glyphosate, while the maize plants of control group were sprayed with water. Then time-series visible/near-infrared hyperspectral images of alive maize plants were collected at 2, 4, 6, and 8 days after treatment (DAT) by a line-scan hyperspectral imaging system. The sample size of each experiment was given in unit of maize plant.

First of all, it is worth noting that the treatment group of R, the control group of R, the treatment group of S, and the control group of S are designated as RT, RW, ST, and SW, respectively. Time-series visible/near-infrared hyperspectral images of alive maize plants were collected at 2, 4, 6, and 8 days after treatment (DAT) by a line-scan HSI system in the visible/near-infrared range (380–1,030 nm), which was reported in detail in previous study (Zhang et al., 2022a). Over the image acquisition, in order to facilitate the extraction of the spectrum of each leaf, it was necessary to ensure that leaves did not overlap with each other and the leaves were as flat as possible. At a distance of 390 mm between the camera lens and the moving sample plate, for the purpose of guaranteeing image quality, the exposure time of camera, the intensity of line light source, and the speed of conveyer belt were adjusted to 70 ms, 240, and 5 mm/s, respectively. Figure 1 shows the detailed steps for the whole experiment.

### Data analysis and model construction

A general processing workflow of hyperspectral data in plant science includes pre-processing, machine learning preparation and model building (Paulus and Mahlein, 2020; Sarić et al., 2022). Therefore, this section explains data analysis according to this workflow.

#### Pre-processing

To eliminate the impact of ambient light, original hyperspectral images needed correcting with a black (selected the camera lens cap with reflectance close to 0) and white reference image (selected the pure white Teflon board with reflectance close to 1). Then, in order to focus on the spectral features of regions of interest and facilitate further analysis, it was essential to identify and segment each leaf in each hyperspectral image and then extract the spectrum of the leaf. This process was divided into two main phases. First, the threshold segmentation method was used to extract the plant region (at 792 nm, the background was separated with a reflectance threshold of 0.1). Second, the stem and leaf were separated by manually selecting the stem region with several rectangles. Based on the shape and reflectance of leaf spectral curve, the abnormal samples caused by measurement errors were rejected. As the study reported (Zhang et al., 2020b), the head-to-tail bands with high noise needs discarding. So only the bands of 450–902 nm were analyzed, and the mean reflectance of all pixels was used to represent the spectral features of corresponding leaf.

Leaf surface reflectance provides a wide perspective for plant growth conditions (Sun et al., 2021). As a derivative index of leaf surface reflectance, the spectral index has been widely used in crop phenotypic monitoring such as stress perception and variety identification (Feng et al., 2019; Shirzadifar et al., 2020b). Consequently, based on the reported literatures (Bergmüller and Vanderwel, 2022; Mushore et al., 2022; Narmilan et al., 2022), sixteen spectral indices related to health status, chemical composition and photosynthesis were selected in this study. Supplementary Table 1 shows the calculation formulas of spectral indices. Then, one-way analysis of variance (ANOVA), followed by the Holm-Bonferroni test (*p* = 0.05) was used to study the feasibility of 16 spectral indices in identifying glyphosate-tolerant cultivar.

#### Machine learning preparation

To prepare the data for modeling, the dataset was divided into two subgroups (training set and test set) with the same feature distribution. The Kennard-Stone (KS) algorithm was used to divide the dataset. KS algorithm selects training dataset samples based on Euclidean distance between variables, and ensures uniform distribution of training dataset samples according to spatial distance (Li et al., 2020). Specifically, samples in the original dataset with the largest distance from the others and as far as possible from the candidate subset are selected to the candidate subset until the division ratio is reached (Morais et al., 2019). For the same dataset, the sample partition results obtained by KS algorithm are the same each time (Chen et al., 2020). Besides, limited by the size of the dataset, the division ratio of the training set and test set was 4:1.

To investigate the transferability of machine learning model in the case of the training set and test set coming from different data distribution, a total of 24 transfer tasks were designed (Supplementary Table 2). In detail, from the perspective of future application, this study took all samples from a single experiment as the source domain dataset, and only the samples from a certain day of another experiment were taken as the target domain dataset.

Considering the data distribution differences between source domain dataset and target domain dataset, in order to further improve the model performance and transferability, two transfer strategies of transfer component analysis (TCA) and source domain updating were used before modeling. On the one hand, as a typical transfer learning algorithm, TCA generally performs the role of preprocessing in data analysis, and its input and output are two large matrices and two small matrices, respectively. TCA maps the source domain dataset and target domain dataset with different distribution to a reproducing kernel Hilbert space, and then continuously reduce the distance between the two domain datasets and retain their internal attributes as many as possible (Panigrahi et al., 2021). Specifically, by exploring an optimal feature map, TCA makes the data distribution of the two domains have the same probability density and the conditional probability density. Maximum mean discrepancy is used to measure the distance between the data distribution of the two domains (Pan et al., 2011; Tao et al., 2019). In this work, primal kernel type was selected and the dimensionality after TCA algorithm was adjusted to 5. On the other hand, the literature (Wan et al., 2020) found that adding partial samples from the new experiment to participate in the model construction upgrades the model performance. In this work, for the convenience of reading, the data update ratio was calculated with reference to the target domain dataset, while the ratio calculated with reference to the source domain dataset was noted in the results and discussion section. In this study, five source domain dataset update levels were set, namely 10, 20, 30, 40, and 50% of target domain (dataset of new experiment).

#### Model building

**FIGURE 2:**
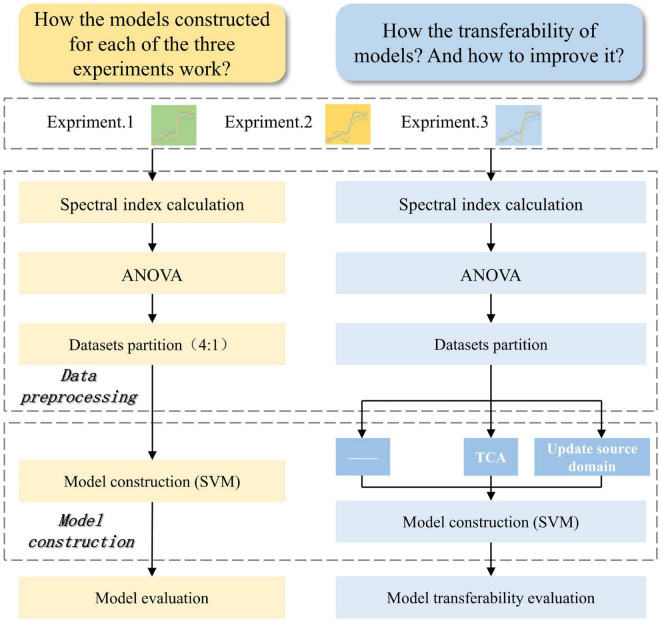
The flowchart of spectral data analysis for glyphosate-resistant cultivar identification. Yellow represents the data flowchart to answer to question that how the models constructed for each of the three experiments work. Blue represents the data flowchart to answer to question that how the transferability of models and how to improve it. ANOVA, analysis of variance. SVM, support vector machine. TCA, transfer component analysis.

As a ubiquitous means of solving high-dimensional datasets, support vector machine (SVM) algorithm is one of the most robust and accurate discrimination methods. From the geometric point of view, the merit of SVM is reflected in the maximum margin needed when constructing hyperplane decision boundaries, so there is sufficient space between interval boundaries to contain test samples. For linear SVM, the general function of the decision boundary is ω^•^ *x* + *b* = 0, where ω is an n-dimensional vector (n is the number of the features), *x* is the data of sample, and *b* is a constant. More detailed theories of SVM algorithm are available in the literature (Ding et al., 2008; Gao and Sun, 2010; Sun et al., 2020). In this work, the *fitcsvm* function in the machine learning toolbox of MATLAB was used to train linear kernel SVM model. In the modeling without any transfer strategy, because the value range of different spectral indices varied greatly, set “standardize” in the function input argument to true. While it was set to false in the modeling with transfer strategy. The reasons were as follows. TCA algorithm could handle such dataset and transfer it into lower dimensional features. After data updating, standardization was considered unsuitable and unreasonable because the new source domain was composed of two sub datasets with different feature distributions. According to the prediction accuracy and training time of the SVM model (relevant data are not presented in this paper), compared with no parameter optimization, automatic parameter optimization greatly increased the training time (685–1304 times), and did not improve model performance (0.86–1.10 times for accuracy) significantly. This result supports the opinion that linear SVM model is not very sensitive to its hyperparameter (Maros et al., 2020). Therefore, the model was trained with the default value kernel parameter in this study. Figure 2 shows the analysis scheme of spectral data of three experiments.

### Model evaluation indices

**FIGURE 3:**
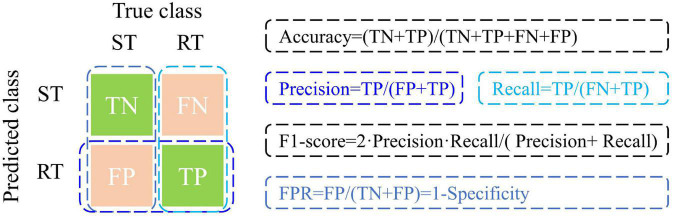
Confusion matrix and statistic formulas for decision model performance evaluation. RT represents resistant cultivar with glyphosate treated. ST represents sensitive cultivar with glyphosate treated. RT plants and ST plants are set as positives and negatives, respectively. TP, FP, TN, and FN represent the number of true positives, false positives, true negatives, false negatives, respectively. The formulas of model performance evaluation parameters are given on the right side of the picture. False positive rate (FPR) indicates the proportion of negative samples incorrectly identified.

To quantitatively evaluate classification model performance, the statistical indices in Figure 3 were calculated. TP, FP, TN, and FN represented the number of true positives, false positives, true negatives, false negatives, respectively. False positive rate (FPR) indicated the proportion of negative samples incorrectly identified. In this work, RT plants and ST plants were set as positives and negatives, respectively.

### Software tools

Stem and leaf segmentation, model construction, and model performance calculation were processed in MATLAB R2016a (Math Works, Natick, MA, United States). All of the graphs were designed by using Origin 2021b (Origin Lab Corporation, Northampton, MA, United States) and Microsoft PowerPoint 2016.

## Results and discussion

### Descriptive statistics of spectral indices

In order to investigate the feasibility of identifying glyphosate-resistant cultivars with 16 spectral indices selected in this work, ANOVA was used to compare each spectral index among four groups (RT, RW, ST, SW) at each sampling time point (2, 4, 6, 8 DAT) in each experiment (Exp.1, Exp.2, Exp.3). Supplementary Table 3 shows the descriptive statistics results of 16 spectral indices.

It can be seen from the descriptive statistics of 16 spectral indices at 2, 4, 6 and 8 DAT in Exp.1 (Supplementary Table 3), at 2 DAT, there was no significant difference (*p* > 0.05) between RT and ST, while at 8 DAT, the difference in most spectral indices of RT and ST were more pronounced (*p* < 0.05).

At 6 DAT, 11 spectral indices of RT and ST showed significant difference (*p* < 0.05). At 8 DAT, EVI (enhanced vegetation index), NRI (nitrogen reflectance index), RDVI (renormalized difference vegetation index) and TCARI/OSAVI (the ratio of transformed chlorophyll absorption in reflectance index to optimized soil-adjusted vegetation index) of RT and ST exhibited pronounced differences for the first time. Although TVI (triangular vegetation index) of RT and ST showed no significant difference up to 8 DAT, it showed significant difference at 6 DAT (Supplementary Table 3) in Exp.3. In addition, according to ANOVA results, there was no significant difference between RT, RW, and SW in every sampling time point, which indicated glyphosate had little effect on R owing to the expression of resistance gene. On the other hand, the significant difference between the two treatment groups (RT and ST) confirmed the feasibility of identifying glyphosate-resistant cultivar based on the selected spectral indices, which contributed to the model development.

### Classification model established on individual experiment

Based on the dataset of the selected spectral indices of glyphosate treatment groups (RT and ST), SVM algorithm was used to evaluate the model performance of each experiment at each sampling time point. For each dataset, confusion maps of classification results on training set and test set were shown in Supplementary Table 4, and the performance evaluation indices were shown in Table 1.

**TABLE 1:** Prediction results of support vector machine models in identifying glyphosate resistant cultivar.

Experiment	Sampling time point	Training set					Test set				
			
		Accuracy_c_	Precision_c_	Recall_c_	F1-score_c_	FPR_c_	Accuracy_p_	Precision_p_	Recall_p_	F1-score_p_	FPR_p_
Exp.1	2–8DAT	0.73	0.73	0.84	0.78	0.41	0.79	0.82	0.69	0.75	0.13
	2DAT	0.86	0.91	0.77	0.83	0.06	0.71	0.75	0.75	0.75	0.33
	4DAT	0.72	0.71	0.83	0.77	0.43	0.57	0.50	0.33	0.40	0.25
	6DAT	0.80	0.82	0.82	0.82	0.23	0.86	1	0.75	0.86	0
	8DAT	0.96	0.94	1	0.97	0.11	1	1	1	1	0
Exp.2	2-8DAT	0.81	0.80	0.93	0.86	0.39	0.85	0.76	1	0.87	0.29
	2DAT	0.75	0.79	0.79	0.79	0.31	0.14	0	0	NaN	0.80
	4DAT	0.88	0.85	0.94	0.89	0.21	0.57	0.50	0.67	0.57	0.50
	6DAT	0.97	0.95	1	0.97	0.08	1	1	1	1	0
	8DAT	1	1	1	1	0	0.75	0.67	1	0.80	0.50
Exp.3	2–8DAT	0.70	0.69	0.94	0.79	0.71	0.84	0.70	0.88	0.78	0.18
	2DAT	0.69	0.68	0.81	0.74	0.44	0.33	0.20	0.33	0.25	0.67
	4DAT	0.95	1	0.90	0.95	0	1	1	1	1	0
	6DAT	1	1	1	1	0	1	1	1	1	0
	8DAT	0.96	0.96	1	0.98	0.17	0.71	0.50	1	0.67	0.40

DAT, days after glyphosate treatment. Exp.1, Exp.2, and Exp.3 represent three independent experiments. The subscripts c and p represent training set and test set respectively.

When the treatment days were not distinguished, the average of accuracy, precision, recall, F1-score, and FPR of SVM models on the test set of three experiments were 0.83, 0.76, 0.86, 0.80, and 0.20, respectively. Among them, three experiments showed significant difference in the recall values, varied from 0.69 to 1. When the dataset obtained on each sampling day was modeled separately, the difference between experiments were even more pronounced. Specifically, for Exp.1, RT was identified correctly at 6 DAT without misjudging ST as RT (FPR = 0 in test set), and the accuracy was 100% at 8 DAT; for Exp.2, the accuracy in test set as early as 6 DAT was 100%; for Exp.3, the SVM model was able to accurately classify RT as early as 4 DAT (accuracy = 1 in test set). The results demonstrated that the earliest accurate identification time of the SVM model may vary with different experiments. It was worth noting that both in Exp.2 and Exp.3, the performance of SVM model at 8 DAT inferior to that at 6 DAT, which was mainly reflected in the misjudgment of ST as RT. Those results may be attributed it to small size of dataset and the fact that some old leaves came close to death no matter what cultivar on 8 DAT. In conclusion, the combination of the selected 16 spectral indices and SVM algorithm could rapidly identify glyphosate resistant and sensitive cultivar in a non-destructive manner.

### Classification model with transfer learning task

According to the results in Table 1 and Supplementary Table 4, the SVM model performance of different experiments varied greatly. So, in this case, how about the transferability of SVM model? Therefore, this section studies the transferability of glyphosate resistant cultivar identification model between different experiments with the 24 transfer learning tasks described in Supplementary Table 2 based on 16 spectral indices.

#### The performances of support vector machine models on transfer learning tasks

As the benchmark for evaluating the performance of transfer strategies, the SVM algorithm was also conducted in 24 transfer learning tasks represented in Supplementary Table 2, and the results on target domain were showed in Supplementary Table 5 and Table 2. The transfer tasks, Exp.1→Exp.2, Exp.1→Exp.3, and Exp.2→Exp.3, performed the best, and the results of SVM model were the same as those of the individual experiment. The two cultivars could be classified accurately at 6 DAT (the confusion matrixes were showed in green). The model performance of the transfer tasks, Exp.2→Exp.1 and Exp.3→Exp.2, was slight worse (the confusion matrixes were showed in blue). On transfer task Exp.2→Exp.1, ST could be correctly recognized at 6 DAT (FPR = 0), but the accuracy of RT (recall) was just 0.19. Transfer task Exp.3→Exp.2 exhibited the best identification result, and the obtained accuracy, precision, recall, F1-score, and FPR were 0.89, 1, 0.81, 0.90, and 0 respectively. All the misclassifications at this time were misjudged RT as ST, which may be because these four samples were about to age completely. The model performance of the transfer task, Exp.3→Exp.1, was the worst (the confusion matrixes were showed in orange), especially for RT recognition. From 2 DAT to 8 DAT, the range of recall was 0.01∼0.29, which was too low to classify accurately. Besides, the SVM model constructed based on Exp.3 had the worst transferability.

**TABLE 2:** Prediction results of support vector machine models on target domain.

Transfer learning task		Target domain					Transfer learning task		Target domain				
			
		Acc.	Pre.	Rec.	F1.	FPR			Acc.	Pre.	Rec.	F1.	FPR
Exp.1→Exp.2	2DAT	0.54	0.54	1	0.70	1	Exp.2→Exp.3	2DAT	0.56	0.54	0.92	0.68	0.79
	4DAT	0.77	0.71	0.95	0.82	0.44		4DAT	0.58	0.56	0.83	0.67	0.67
	6DAT	0.95	0.91	1	0.95	0.13		6DAT	1	1	1	1	0
	8DAT	1	1	1	1	0		8DAT	0.91	1	0.88	0.93	0
Exp.1→Exp.3	2DAT	0.50	0.50	1	0.67	1	Exp.3→Exp.1	2DAT	0.64	0.83	0.29	0.43	0.05
	4DAT	0.50	0.50	1	0.67	1		4DAT	0.51	0.67	0.19	0.30	0.11
	6DAT	1	1	1	1	0		6DAT	0.49	1	0.10	0.17	0
	8DAT	1	1	1	1	0		8DAT	0.45	1	0.11	0.20	0
Exp.2→Exp.1	2DAT	0.58	0.75	0.18	0.29	0.05	Exp.3→Exp.2	2DAT	0.59	0.63	0.57	0.60	0.39
	4DAT	0.59	0.65	0.52	0.58	0.33		4DAT	0.64	0.63	0.81	0.71	0.56
	6DAT	0.54	1	0.19	0.32	0		6DAT	0.89	1	0.81	0.89	0
	8DAT	0.91	1	0.88	0.93	0		8DAT	0.79	1	0.76	0.86	0

Acc., accuracy; Pre., precision; Rec., recall; F1., F1-score; FPR, False Positive Rate; DAT, days after glyphosate treatment. Exp.1, Exp.2, and Exp.3 represent three independent experiments.

Compared to Table 1, the difference in data distribution between the source domain and the target domain weakened the performance of SVM models to varying degrees. Furthermore, the classification accuracy and transferability of SVM models were different with the source domain. Overall, although the SVM algorithm had the potential to transfer between experiments carried out at different times, the dependence on specific transfer tasks resulted in the low stability of transferability. Therefore, it was necessary to further explore whether there were solutions to improve the transferability of glyphosate tolerance discrimination models between different experiments.

#### The performances of transfer component analysis_support vector machine models on transfer learning tasks

**FIGURE 4:**
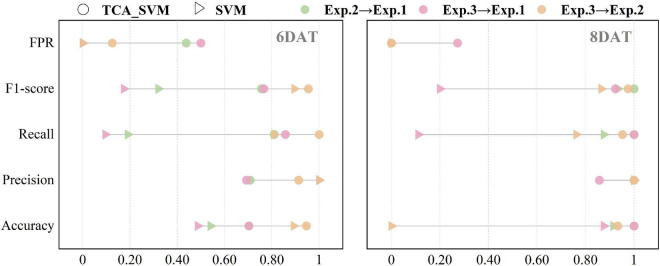
Prediction results of support vector machine models in target domain before and after using transfer component analysis algorithm. TCA_SVM, transfer component analysis-based support vector machine model.

For the three transfer tasks (Exp.2→Exp.1, Exp.3→Exp.1, Exp.3→Exp.2) with poor classification accuracy in section “The performances of SVM models on transfer learning tasks,” TCA algorithm was applied in an attempt to improve the transferability of SVM models. After narrowing the data distribution distance difference between source domain and target domain, the SVM algorithm was applied to develop models using the five transformed features of source domain, and the results were showed in Supplementary Table 6 and Table 3. Among them, the TCA_SVM model of Exp.3→Exp.2 obtained the best performance. At 6 DAT, accuracy, precision, recall, F1-score, and FPR were 95, 91, 100%, 0.95 and 13% respectively on target source. The discrimination accuracy basically reached the level of SVM model constructed based on a single experiment, and the confusion maps were showed in green. Compared to SVM models, for the transfer tasks, Exp.2→Exp.1 and Exp.3→Exp.1, TCA_SVM models improved the performance indices of accuracy, recall, and F1-score on target domain (Figure 4), which to some extent solved the problem of misclassifying RT to ST in SVM models. However, instead of improving, the performance indices of precision and FPR even went in the opposite direction. Specifically, it resulted in the misjudgment of ST as RT (Supplementary Table 6), which should be avoided in the screening process of resistant cultivars compared with misjudgment of RT as ST. Therefore, for the transfer learning tasks, Exp.2→Exp.1 and Exp.3→Exp.1, it was necessary to further explore other transfer learning strategy to optimize the transferability of SVM models.

**TABLE 3:** Prediction results of transfer component analysis-based support vector machine models on target domain.

Transfer learning task		Target domain				
	
		Accuracy	Prediction	Recall	F1-score	FPR
Exp.2→Exp.1	2DAT	0.47	0.46	0.71	0.56	0.74
	4DAT	0.56	0.57	0.81	0.67	0.72
	6DAT	0.70	0.71	0.81	0.76	0.44
	8DAT	1	1	1	1	0
Exp.3→Exp.1	2DAT	0.44	0.44	0.71	0.55	0.79
	4DAT	0.54	0.55	0.81	0.65	0.78
	6DAT	0.70	0.69	0.86	0.77	0.50
	8DAT	0.90	0.86	1	0.92	0.27
Exp.3→Exp.2	2DAT	0.56	0.55	1	0.71	0.94
	4DAT	0.54	0.54	1	0.70	1
	6DAT	0.95	0.91	1	0.95	0.13
	8DAT	0.96	1	0.95	0.98	0

DAT, days after glyphosate treatment. Exp.1, Exp.2, and Exp.3 represent three independent experiments.

#### The performances of Update_support vector machine models on transfer learning tasks

After TCA applied, although the classification accuracy of the transfer tasks (Exp.2→Exp.1 and Exp.3→Exp.1) improved, it was still failed to reach the level of SVM models based on a single experiment. Therefore, did transfer learning strategy_2 (update source domain) performed better in improvement of SVM transferability?

In this work, five source domain dataset updating levels were set, namely 10, 20, 30, 40, and 50% of target domain. Supplementary Table 7 and Table 4 show the results of Update_SVM models on new target domains. In general, consistent with the literature reported (Weng et al., 2018; Wan et al., 2020), the performance of Update_SVM models are improved with the increase of the proportion of new samples in source domain. For the transfer learning task Exp.2→Exp.1, when 50% of Exp.1_6DAT dataset (13% of the source domain) samples were randomly selected and added into the source domain, accuracy of Update_SVM model on target domain reached 78% (increased by 44%), recall increased from 19 to 56%, and F1-score increased from 0.32 to 0.71. When classifying samples of Exp.1_8DAT, 100% accuracy could be achieved by adding only 10% new samples from target domain (2% of the source domain). For the transfer learning task Exp.3→Exp.1, when 40% of Exp.1_6DAT samples (11% of the source domain) were added to the source domain, accuracy of Update_SVM model on target domain reached 77% (increased by 59%), recall increased from 10 to 73%, and F1-score increased from 0.17 to 0.76. When classifying samples of Exp.1_8DAT, the best result appeared when 30% new samples were added, where accuracy, precision, recall, F1-score and FPR were 75, 100, 62%, 0.76 and 0, respectively. Compared with the performance of SVM model based on a single experiment, there was still obvious improvement room.

**TABLE 4:** Prediction results of source domain updating-based support vector machine models on target domain.

Transfer learning task		Ratio^a^ (%)	Target domain				
		
			Accuracy	Prediction	Recall	F1-score	FPR
Exp.2→Exp.1	6DAT	10	0.55	0.83	0.26	0.40	0.07
		20	0.59	1	0.25	0.40	0
		30	0.64	1	0.31	0.47	0
		40	0.64	1	0.27	0.43	0
		50	0.78	1	0.56	0.71	0
	8DAT	10	1	1	1	1	0
		20	1	1	1	1	0
		30	1	1	1	1	0
		40	1	1	1	1	0
		50	1	1	1	1	0
Exp.3→Exp.1	6DAT	10	0.64	0.89	0.42	0.57	0.07
		20	0.69	0.77	0.63	0.69	0.23
		30	0.72	0.80	0.62	0.70	0.17
		40	0.77	0.80	0.73	0.76	0.18
		50	0.72	0.75	0.67	0.71	0.22
	8DAT	10	0.62	1	0.41	0.58	0
		20	0.70	1	0.53	0.70	0
		30	0.75	1	0.62	0.76	0
		40	0.76	1	0.60	0.75	0
		50	0.71	1	0.43	0.60	0

^a^Source domain dataset update levels were set, namely 10, 20, 30, 40, and 50% of target domain. DAT, days after glyphosate treatment. Exp.1, Exp.2, and Exp.3 represent three independent experiments.

Compared with direct transfer (Table 2 and Supplementary Table 5), TCA algorithm and source domain updating strategies greatly improved the prediction accuracy, recall, and F1-score. But the former had a higher FPR value, and led to an increase in the proportion of ST misjudged as RT in detection, which was the least expected misjudgment in breeding screening. For transfer learning task Exp.3→Exp.1_8DAT, TCA algorithm worked better than source domain updating strategy. Therefore, both the two strategies had similar improvement on the transferability of SVM model in different datasets of experiment. Can the two transfer strategies be applied simultaneously to achieve better results?

#### The performances of Update_TCA_support vector machine models on transfer learning task

Since the performance of TCA_SVM and Update_SVM model in the transfer learning task Exp.3→Exp.1 was quite different, this section explores the question of whether the two transfer strategies could complement each other’s advantages to further improve the performance of the SVM model.

**FIGURE 5:**
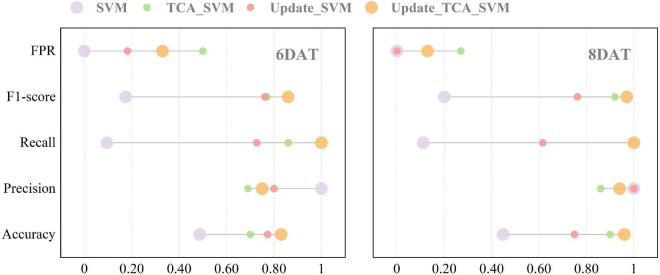
Performance of four models on new target domain in transfer learning task (Exp.3→Exp.1). SVM, support vector machine model. TCA_SVM, transfer component analysis-based support vector machine model. Update_SVM, source domain updating-based support vector machine model. Update_TCA_SVM, source domain updating- and transfer component analysis-based support vector machine model.

Figure 5 and Table 5 detail the classification results of the Update_TCA_SVM models on new target domain in transfer learning task Exp.3→Exp.1. When 50% of Exp.1_6DAT dataset (14% of the source domain) samples were randomly selected and added into the source domain, accuracy of Update_TCA_SVM model on target domain reached 83% (increased by 71%), recall increased from 10 to 100%, and F1-score increased from 0.17 to 0.86. When classifying samples of Exp.1_8DAT, the best result appeared when 20% new samples (5% of the source domain) were added, where accuracy, precision, recall, F1-score and FPR were 96, 94, 100%, 0.97 and 13%, respectively. Among them, classification accuracy, recall and F1-score were significantly higher than those in SVM, TCA_SVM and Update_SVM models (Supplementary Table 8). Moreover, source domain updating strategy had a weakening effect on the increase of FPR value brought by TCA algorithm. The two transfer strategies could complement each other’s advantages to achieve the best transferability and model performance.

**TABLE 5:** Prediction results of transfer component analysis- and source domain updating-based support vector machine models on new target domain in transfer learning task (Exp.3→Exp.1).

Transfer learning task		Ratio^a^ (%)	Target domain				
		
			Accuracy	Prediction	Recall	F1-score	FPR
Exp.3→Exp.1	6DAT	10	0.73	0.73	0.84	0.78	0.43
		20	0.72	0.72	0.81	0.76	0.38
		30	0.80	0.72	1	0.84	0.42
		40	0.82	0.73	1	0.85	0.36
		50	0.83	0.75	1	0.86	0.33
	8DAT	10	0.92	0.94	0.94	0.94	0.11
		20	0.96	0.94	1	0.97	0.13
		30	0.95	0.93	1	0.96	0.14
		40	0.88	0.83	1	0.91	0.29
		50	0.86	0.78	1	0.88	0.29

^a^Source domain dataset update levels were set, namely 10, 20, 30, 40, and 50% of target domain. DAT, days after glyphosate treatment. Exp.1, Exp.2, and Exp.3 represent three independent experiments.

## Discussion

### Potential implementation of spectral index for the filed detection

The merits of glyphosate in field management promote the creation of glyphosate resistant cultivars. Planting is an important step to verify the glyphosate tolerance of the new cultivars developed through genetic engineering and other technologies. Visual observation is still the mainstream method for breeders to identify glyphosate resistant cultivars (Singh et al., 2021), which usually takes several weeks and is time-consuming and laborious, severely limiting the breeding process. The difference between resistant and sensitive cultivars is that the response of the latter to glyphosate stress is more easily observed than that of the former (Shirzadifar et al., 2020b). Glyphosate affects the photosynthetic activity of plants by inhibiting the shikimic acid pathway (Gomes et al., 2014), which is eventually reflected in leaf surface reflectance. At present, hyperspectral technology has been widely used in the early detection of stress due to its high throughput, rapid and non-destructive nature (Sun et al., 2021; Sarić et al., 2022). Visible near infrared spectroscopy can capture the changes in leaf reflectance in time so as to realize the identification of resistance cultivars. However, it should be pointed out that the high dimension of spectral data limits the calculation speed to some extent, while the spectral index is a combination of several bands, which can obtain similar results while reducing the dimension (Bloem et al., 2020). In this work, living plants were used to achieve non-destructive identification, which was different from *in vitro* leaves reported in previous study (Zhang et al., 2022a). The model constructed based on spectral index could accurately classify glyphosate resistant cultivars at 6 DAT (accuracy = 100% in Table 1), which was higher than previous study (Feng et al., 2018), indicating the feasibility and effectiveness of spectral index in the identification of glyphosate resistant cultivar, and the detection performance was better than the sensitive wavelengths and sensitive chlorophyll fluorescence parameters.

**FIGURE 6:**
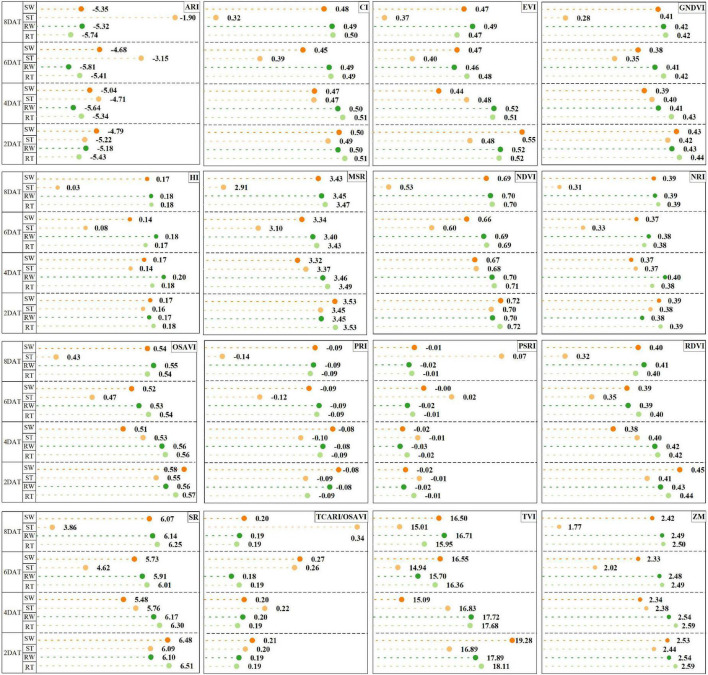
Time-series effect of glyphosate on the responses of leaf spectral indices at 2, 4, 6, and 8 days after treatment (DAT) in Experiment 1. The spectral index value is presented as means. Letters highlight significant difference among four groups (*p* < 0.05) by the Holm-Bonferroni test.

Many studies (Zhang et al., 2019; Zea et al., 2022) have been emphasized the importance of spectral index in cultivars identification and early detection of stress. In this work, at 6 DAT, most spectral indices, such as ARI (anthocyanin reflectance index), PRI (photochemical reflectance index) and PSRI (plant senescence reflectance index), were able to detect differences between RT and ST. ARI is sensitive to anthocyanin in leaves, and the larger ARI value is, the closer the plant is to death (Gitelson et al., 2006). Owing to weak defense system to glyphosate, S plants withed gradually over time with glyphosate treatment. PRI is sensitive to carotenoids in living plants, and used to evaluate the utilization efficiency of incident light by plant in photosynthesis, which is directly related to carbon absorption efficiency, plant growth rate and photosynthetically active radiation (Gamon et al., 1992; Peñuelas et al., 1995). Hence, PRI can be used to study vegetation productivity and stress, senescence of crops. As Supplementary Table 3 and Figure 6 show, glyphosate accelerated the aging of S plants exhibited higher PRI values, but had no significant effect on R plants. Besides, previous study (Huang et al., 2016) reported that the soybean sprayed with herbicide can be accurately distinguished from the control plants at an early stage based on the spectral index analysis, especially ARI and PRI, which is consistent with the results of our research. As another spectral index associated with plant senescence, PSRI is sensitive to the ratio of carotenoids to chlorophyll in living plants and its increase is often related to changes in physiological and phenological status due to plant stresses (Merzlyak et al., 1999; Yu et al., 2018). Driven by glyphosate treatment and low tolerance, carotenoids and chlorophyll content in S plant leaves gradually increased and decreased respectively, so PSRI values were higher than other groups. Here, significant changes in spectral indices were associated with severity of stress development on leaves of S plants, which led to decreased photosynthetic activities, distinct the senescence signatures and stunted growth. The above results are consistent with other studies (da Silva Santos et al., 2020; Hassannejad et al., 2020). Although the stress in different researches was different, the physiological changes caused by stress were similar. Hence, the spectral index could be applied to the early detection of various stresses and the identification of target cultivars.

### Transfer strategy improves model performance in different experiments

Due to the difference in data feature distribution between the historical dataset and the new dataset, the model constructed by the historical dataset with traditional modeling algorithms may be invalid when predicting the sample spectra of different experiments, which is shown in the section “The performances of SVM models on transfer learning tasks” (Qiu et al., 2020; Zhao et al., 2021). Supplementary Table 9 shows the original datasets of three experiments. Transfer learning can help the model transfer the knowledge learned from the source domain to the target domain and reduce the adverse impact of data distribution differences on model performance (Cheplygina et al., 2019; Talo et al., 2019).

According to the result of ANOVA (Figure 6 and Supplementary Table 3), the spectral indices of RT and ST were significantly different at 6 DAT at the earliest, which was consistent with the modeling results (Table 1 and Supplementary Table 4) of the single experiment. Here, how to accurately identify glyphosate resistant cultivar at 6 DAT in transfer tasks was one of the primary goals in this work. Hence, two transfer learning strategies including the TCA algorithm and source domain updating, were used to improve model performance in identifying the new samples from different experiments. And the model with transfer learning strategies could also accurately identify resistant cultivar at 6 DAT in most transfer tasks. The two transfer strategies could complement each other’s advantages to achieve the best transferability and model performance. For the transfer learning task (Exp.3→Exp.1) with the worst classification results, when 50% of Exp.1_6DAT dataset (14% of the source domain) samples were randomly selected and added into the source domain (Figure 5 and Tables 2, 5), compared with the SVM model, the accuracy of Update_TCA_SVM model on target domain reached 83% (increased by 71%), recall increased from 10 to 100%, and F1-score increased from 0.17 to 0.86. Previous literature (Tao et al., 2019) reported that the transfer model can achieve an effective prediction by collecting the current samples to the training set, which is consistent with the results of our research. Their results also pointed out that the prediction accuracy of transfer model can be further improved by using more current samples. However, our results (Tables 4, 5) are not consistent with it, which may be due to saturation of the number of adding samples. Anyway, the distinct improvement of the model transferability prompts us to further explore the universality of the two transfer learning strategies applied in this work in more scenarios.

### Potential applications and future prospect

Based on the selected spectral indices in our study, portable sensors should be developed and integrated with transfer learning algorithms in the near future. Then attaching these sensors to unmanned aerial vehicle to realize the rapid and non-destructive identification of target cultivars at the field or regional scale. Moreover, in order to study the universality of the transfer learning strategy, it is suggested to collect more different samples in more growing environments and cultivars.

## Conclusion

In this study, the HSI system was used to obtain hyperspectral image of samples, and after stem and leaf segmentation, the mean spectra and 16 spectral indices of each leaf was calculated. Then transfer learning strategies were implemented to construct a model for identifying glyphosate-resistant cultivars in different experiments. As one of the classification models, SVM algorithm was employed to explore the model transferability between different experiments, and assessed the effectiveness of two transfer learning strategies including TCA algorithm and source domain updating. For one of the transfer tasks, transferability of SVM model was improved by randomly selecting 14% of source domain from target domain to train and applying transfer component analysis algorithm, the accuracy on target domain reached 83% (increased by 71%), recall increased from 10 to 100%, and F1-score increased from 0.17 to 0.86. The overall results indicated that compared with direct model transfer, both transfer learning strategies improved model transferability between different experiments although the prediction results varied with different added number of new samples from source domain, and these two strategies could complement each other’s advantages. Inspired by the distinct positive contribution of the transfer learning strategy, future work will be concentrated on experiments with more cultivars, growing conditions and spectral devices to investigate the universality of the transfer learning strategies. Ideally, these results could someday be validated and optimized enough that ultra-portable instrument combined with the transfer learning strategy could be employed to screen glyphosate resistant cultivars on large scale in a rapid, non-destructive and high-throughput way, which could help breeders improve work efficiency.

## Data availability statement

The original contributions presented in this study are included in the article/Supplementary material, further inquiries can be directed to the corresponding author/s.

## Author contributions

MT and XF conceived, designed the experiments, analyzed the data, and wrote the manuscript. MT, XB, and XC performed the experiments. XF, YH, YW, and CP made critical comments and revisions. All authors contributed to the article and approved the submitted version.

## References

[B1] An D. Zhang L. Liu Z. Liu J. Wei Y. (2022). Advances in infrared spectroscopy and hyperspectral imaging combined with artificial intelligence for the detection of cereals quality. *Crit. Rev. Food Sci. Nutr.* 20 1–31. 10.1080/10408398.2022.2066062 35442834

[B2] Bergmüller K. O. Vanderwel M. C. (2022). Predicting tree mortality using spectral indices derived from multispectral UAV imagery. *Remote Sens.* 14:2195. 10.3390/rs14092195

[B3] Bloem E. Gerighausen H. Chen X. Schnug E. (2020). The potential of spectral measurements for identifying glyphosate application to agricultural fields. *Agronomy* 10:1409. 10.3390/agronomy10091409

[B4] Chen X. Dong Z. Liu J. Wang H. Zhang Y. Chen T. (2020). Hyperspectral characteristics and quantitative analysis of leaf chlorophyll by reflectance spectroscopy based on a genetic algorithm in combination with partial least squares regression. *Spectrochim. Acta A Mol. Biomol. Spectrosc.* 243:118786. 10.1016/j.saa.2020.118786 32854083

[B5] Cheplygina V. de Bruijne M. Pluim J. P. W. (2019). Not-so-supervised: A survey of semi-supervised, multi-instance, and transfer learning in medical image analysis. *Med. Image Anal.* 54 280–296. 10.1016/j.media.2019.03.009 30959445

[B6] Clapp J. (2021). Explaining growing glyphosate use: The political economy of herbicide-dependent agriculture. *Glob. Environ. Chang.* 67:102239. 10.1016/j.gloenvcha.2021.102239

[B7] Corrêa E. A. Dayan F. E. Owens D. K. Rimando A. M. Duke S. O. (2016). Glyphosate-resistant and conventional canola (*Brassica napus* L.) responses to glyphosate and Aminomethylphosphonic Acid (AMPA) treatment. *J. Agric. Food Chem.* 64 3508–3513. 10.1021/acs.jafc.6b00446 27092715

[B8] da Silva Santos J. da Silva Pontes M. Grillo R. Fiorucci A. R. José de Arruda G. Santiago E. F. (2020). Physiological mechanisms and phytoremediation potential of the macrophyte Salvinia biloba towards a commercial formulation and an analytical standard of glyphosate. *Chemosphere* 259:127417. 10.1016/j.chemosphere.2020.127417 32623201

[B9] Ding Y. Song X. Zen Y. (2008). Forecasting financial condition of Chinese listed companies based on support vector machine. *Exp. Syst. Appl.* 34 3081–3089. 10.1016/j.eswa.2007.06.037

[B10] Duke S. O. Powles S. B. (2008). Glyphosate: A once-in-a-century herbicide. *Pest Manag. Sci.* 64 319–325. 10.1002/ps.1518 18273882

[B11] Feng X. Chen H. Chen Y. Zhang C. Liu X. Weng H. (2019). Rapid detection of cadmium and its distribution in *Miscanthus sacchariflorus* based on visible and near-infrared hyperspectral imaging. *Sci. Total Environ.* 659 1021–1031. 10.1016/j.scitotenv.2018.12.458 31096318

[B12] Feng X. Yu C. Chen Y. Peng J. Ye L. Shen T. (2018). Non-destructive determination of shikimic acid concentration in transgenic maize exhibiting glyphosate tolerance using chlorophyll fluorescence and hyperspectral imaging. *Front. Plant Sci.* 9:468. 10.3389/fpls.2018.00468 29686693 PMC5900420

[B13] Gamon J. A. Peñuelas J. Field C. B. (1992). A narrow-waveband spectral index that tracks diurnal changes in photosynthetic efficiency. *Remote Sens. Environ.* 41 35–44. 10.1016/0034-4257(92)90059-S

[B14] Gao Y. Sun S. (2010). “An empirical evaluation of linear and nonlinear kernels for text classification using support vector machines,” in *Proceedings of the–2010 7th international conference on fuzzy systems and knowledge discovery, FSKD 2010*, (Yantai: IEEE), 1502–1505. 10.1109/FSKD.2010.5569327

[B15] Gitelson A. A. Keydan G. P. Merzlyak M. N. (2006). Three-band model for noninvasive estimation of chlorophyll, carotenoids, and anthocyanin contents in higher plant leaves. *Geophys. Res. Lett.* 33 2–6. 10.1029/2006GL026457

[B16] Gomes M. P. Smedbol E. Chalifour A. Hénault-Ethier L. Labrecque M. Lepage L. (2014). Alteration of plant physiology by glyphosate and its by-product aminomethylphosphonic acid: An overview. *J. Exp. Bot.* 65 4691–4703. 10.1093/jxb/eru269 25039071

[B17] Greener J. G. Kandathil S. M. Moffat L. Jones D. T. (2022). A guide to machine learning for biologists. *Nat. Rev. Mol. Cell Biol.* 23 40–55. 10.1038/s41580-021-00407-0 34518686

[B18] Gu Q. Sheng L. Zhang T. Lu Y. Zhang Z. Zheng K. (2019). Early detection of tomato spotted wilt virus infection in tobacco using the hyperspectral imaging technique and machine learning algorithms. *Comput. Electron. Agric.* 167:105066. 10.1016/j.compag.2019.105066

[B19] Hassannejad S. Lotfi R. Ghafarbi S. P. Oukarroum A. (2020). Early identification of herbicide modes of action by the use of chlorophyll fluorescence measurements. *Plants* 9:529. 10.3390/plants9040529 32325997 PMC7238274

[B20] Huang Y. Yuan L. Reddy K. N. Zhang J. (2016). In-situ plant hyperspectral sensing for early detection of soybean injury from dicamba. *Biosyst. Eng.* 149 51–59. 10.1016/j.biosystemseng.2016.06.013

[B21] Li T. Fong S. Wu Y. Tallón-Ballesteros A. J. (2020). “Kennard-Stone balance algorithm for time-series big data stream mining,” in *Proceedings of the 2020 international conference on data mining workshops*, (Sorrento, IT: ICDMW), 851–858. 10.1109/ICDMW51313.2020.00122

[B22] Liang K. Huang J. He R. Wang Q. Chai Y. Shen M. (2020). Comparison of Vis-NIR and SWIR hyperspectral imaging for the non-destructive detection of DON levels in Fusarium head blight wheat kernels and wheat flour. *Infrared Phys. Technol.* 106 103281. 10.1016/j.infrared.2020.103281

[B23] Lin W. Zhang Z. Chen Y. Zhang Q. Ke M. Lu T. (2023). The mechanism of different cyanobacterial responses to glyphosate. *J. Environ. Sci.* 125 258–265. 10.1016/j.jes.2021.11.039 36375911

[B24] Lu X. Zhang S. Tian Y. Li Y. Wen R. Tsou J. Y. (2020). Monitoring suaeda salsa spectral response to salt conditions in coastal wetlands: A case study in dafeng elk national nature reserve, China. *Remote Sens.* 12:2700. 10.3390/RS12172700

[B25] Maione C. Barbosa F. Barbosa R. M. (2019). Predicting the botanical and geographical origin of honey with multivariate data analysis and machine learning techniques: A review. *Comput. Electron. Agric.* 157 436–446. 10.1016/j.compag.2019.01.020

[B26] Maros M. E. Capper D. Jones D. T. W. Hovestadt V. von Deimling A. Pfister S. M. (2020). Machine learning workflows to estimate class probabilities for precision cancer diagnostics on DNA methylation microarray data. *Nat. Protoc.* 15 479–512. 10.1038/s41596-019-0251-6 31932775

[B27] Merzlyak M. N. Gitelson A. A. Chivkunova O. B. Rakitin V. Y. (1999). Non-destructive optical detection of pigment changes during leaf senescence and fruit ripening. *Physiol. Plant.* 106 135–141. 10.1034/j.1399-3054.1999.106119.x 11841302

[B28] Morais C. L. M. Santos M. C. D. Lima K. M. G. Martin F. L. (2019). Improving data splitting for classification applications in spectrochemical analyses employing a random-mutation Kennard-Stone algorithm approach. *Bioinformatics* 35 5257–5263. 10.1093/bioinformatics/btz421 31116391 PMC6954661

[B29] Mushore T. D. Mutanga O. Odindi J. (2022). Estimating urban LST using multiple remotely sensed spectral indices and elevation retrievals. *Sustain. Cities Soc.* 78:103623. 10.1016/j.scs.2021.103623

[B30] Najafabadi M. Y. (2021). *Using advanced proximal sensing and genotyping tools combined with bigdata analysis methods to improve b soybean yield*. [master’s thesis]. Guelph: University of Guelph.

[B31] Narmilan A. Gonzalez F. Salgadoe A. S. A. Kumarasiri U. W. L. M. Weerasinghe H. A. S. Kulasekara B. R. (2022). Predicting canopy chlorophyll content in sugarcane crops using machine learning algorithms and spectral vegetation indices derived from uav multispectral imagery. *Remote Sens.* 14:1140. 10.3390/rs14051140

[B32] Pan L. Yu Q. Han H. Mao L. Nyporko A. Fan L. J. (2019). Aldo-keto reductase metabolizes glyphosate and confers glyphosate resistance in *Echinochloa colona*. *Plant Physiol.* 181 1519–1534. 10.1104/pp.19.00979 31551360 PMC6878027

[B33] Pan S. J. Tsang I. W. Kwok J. T. Yang Q. (2011). Domain adaptation via transfer component analysis. *IEEE Trans. Neural Netw.* 22 199–210. 10.1109/TNN.2010.2091281 21095864

[B34] Panigrahi S. Nanda A. Swarnkar T. (2021). A survey on transfer learning. *Smart Innov. Syst. Technol.* 194 781–789. 10.1007/978-981-15-5971-6_83

[B35] Paulus S. Mahlein A. K. (2020). Technical workflows for hyperspectral plant image assessment and processing on the greenhouse and laboratory scale. *Gigascience* 9 1–10. 10.1093/gigascience/giaa090 32815537 PMC7439585

[B36] Peñuelas J. Filella I. Gamon J. A. (1995). Assessment of photosynthetic radiation-use efficiency with spectral reflectance. *New Phytol.* 131 291–296. 10.1111/j.1469-8137.1995.tb03064.x

[B37] Qiu Z. Zhao S. Feng X. He Y. (2020). Transfer learning method for plastic pollution evaluation in soil using NIR sensor. *Sci. Total Environ.* 740:140118. 10.1016/j.scitotenv.2020.140118 32559549

[B38] Sarić R. Nguyen V. D. Burge T. Berkowitz O. Trtílek M. Whelan J. (2022). Applications of hyperspectral imaging in plant phenotyping. *Trends Plant Sci.* 27 301–315. 10.1016/j.tplants.2021.12.003 34998690

[B39] Shirzadifar A. Bajwa S. Nowatzki J. Bazrafkan A. (2020a). Field identification of weed species and glyphosate-resistant weeds using high resolution imagery in early growing season. *Biosyst. Eng.* 200 200–214. 10.1016/j.biosystemseng.2020.10.001

[B40] Shirzadifar A. Bajwa S. Nowatzki J. Shojaeiarani J. (2020b). Development of spectral indices for identifying glyphosate-resistant weeds. *Comput. Electron. Agric.* 170:105276. 10.1016/j.compag.2020.105276

[B41] Singh V. Dou T. Krimmer M. Singh S. Humpal D. Payne W. Z. (2021). Raman spectroscopy can distinguish glyphosate-susceptible and –resistant palmer amaranth (*Amaranthus palmeri*). *Front. Plant Sci.* 12:657963. 10.3389/fpls.2021.657963 34149756 PMC8212978

[B42] Sun D. Robbins K. Morales N. Shu Q. Cen H. (2021). Advances in optical phenotyping of cereal crops. *Trends Plant Sci.* 27 191–208. 10.1016/j.tplants.2021.07.015 34417079

[B43] Sun J. Li H. Fujita H. Fu B. Ai W. (2020). Class-imbalanced dynamic financial distress prediction based on Adaboost-SVM ensemble combined with SMOTE and time weighting. *Inf. Fusion* 54 128–144. 10.1016/j.inffus.2019.07.006

[B44] Talo M. Baloglu U. B. Yıldırım Ö Rajendra Acharya U. (2019). Application of deep transfer learning for automated brain abnormality classification using MR images. *Cogn. Syst. Res.* 54 176–188. 10.1016/j.cogsys.2018.12.007

[B45] Tao C. Wang Y. Cui W. Zou B. Zou Z. Tu Y. (2019). A transferable spectroscopic diagnosis model for predicting arsenic contamination in soil. *Sci. Total Environ.* 669 964–972. 10.1016/j.scitotenv.2019.03.186 30970463

[B46] Van Bruggen A. H. C. He M. M. Shin K. Mai V. Jeong K. C. Finckh M. R. (2018). Environmental and health effects of the herbicide glyphosate. *Sci. Total Environ.* 61 255–268. 10.1016/j.scitotenv.2017.10.309 29117584

[B47] Wan L. Cen H. Zhu J. Zhang J. Zhu Y. Sun D. (2020). Grain yield prediction of rice using multi-temporal UAV-based RGB and multispectral images and model transfer–a case study of small farmlands in the South of China. *Agric. For. Meteorol.* 291:108096. 10.1016/j.agrformet.2020.108096

[B48] Weng H. Lv J. Cen H. He M. Zeng Y. Hua S. (2018). Hyperspectral reflectance imaging combined with carbohydrate metabolism analysis for diagnosis of citrus Huanglongbing in different seasons and cultivars. *Sens. Actuators. B. Chem.* 275 50–60. 10.1016/j.snb.2018.08.020

[B49] Weng S. Han K. Chu Z. Zhu G. Liu C. Zhu Z. (2021). Reflectance images of effective wavelengths from hyperspectral imaging for identification of Fusarium head blight-infected wheat kernels combined with a residual attention convolution neural network. *Comput. Electron. Agric.* 190:106483. 10.1016/j.compag.2021.106483

[B50] Yang D. Yuan J. Chang Q. Zhao H. Cao Y. (2020). Early determination of mildew status in storage maize kernels using hyperspectral imaging combined with the stacked sparse auto-encoder algorithm. *Infrared Phys. Technol.* 109:103412. 10.1016/j.infrared.2020.103412

[B51] Yu K. Anderegg J. Mikaberidze A. Karisto P. Mascher F. McDonald B. A. (2018). Hyperspectral canopy sensing of wheat septoria tritici blotch disease. *Front. Plant Sci.* 9:1195. 10.3389/fpls.2018.01195 30174678 PMC6108383

[B52] Zea M. Souza A. Yang Y. Lee L. Nemali K. Hoagland L. (2022). Leveraging high-throughput hyperspectral imaging technology to detect cadmium stress in two leafy green crops and accelerate soil remediation efforts. *Environ. Pollut.* 292:118405. 10.1016/j.envpol.2021.118405 34710518

[B53] Zhang C. Wu W. Zhou L. Cheng H. Ye X. He Y. (2020a). Developing deep learning based regression approaches for determination of chemical compositions in dry black goji berries (*Lycium ruthenicum* Murr.) using near-infrared hyperspectral imaging. *Food Chem.* 319:126536. 10.1016/j.foodchem.2020.126536 32146292

[B54] Zhang J. Feng X. Wu Q. Yang G. Tao M. Yang Y. (2022a). Rice bacterial blight resistant cultivar selection based on visible/near-infrared spectrum and deep learning. *Plant Methods* 18 1–16. 10.1186/s13007-022-00882-2 35428329 PMC9013134

[B55] Zhang J. Huang Y. Reddy K. N. Wang B. (2019). Assessing crop damage from dicamba on non-dicamba-tolerant soybean by hyperspectral imaging through machine learning. *Pest Manag. Sci.* 75 3260–3272. 10.1002/ps.5448 31006969

[B56] Zhang J. Yang Y. Feng X. Xu H. Chen J. He Y. (2020b). Identification of bacterial blight resistant rice seeds using terahertz imaging and hyperspectral imaging combined with convolutional neural network. *Front. Plant Sci.* 11:821. 10.3389/fpls.2020.00821 32670316 PMC7326944

[B57] Zhang T. Huang Y. Reddy K. N. Yang P. Zhao X. Zhang J. (2021b). Using machine learning and hyperspectral images to assess damages to corn plant caused by glyphosate and to evaluate recoverability. *Agronomy* 11 591–596. 10.3390/agronomy11030583

[B58] Zhang L. Sun H. Li H. Rao Z. Ji H. (2021a). Identification of rice-weevil (*Sitophilus oryzae* L.) damaged wheat kernels using multi-angle NIR hyperspectral data. *J. Cereal Sci.* 101:103313. 10.1016/j.jcs.2021.103313

[B59] Zhang L. Wang Y. Wei Y. An D. (2022b). Near-infrared hyperspectral imaging technology combined with deep convolutional generative adversarial network to predict oil content of single maize kernel. *Food Chem.* 370:131047. 10.1016/j.foodchem.2021.131047 34626928

[B60] Zhao S. Qiu Z. He Y. (2021). Transfer learning strategy for plastic pollution detection in soil: Calibration transfer from high-throughput HSI system to NIR sensor. *Chemosphere* 272:129908. 10.1016/j.chemosphere.2021.129908 35534971

[B61] Zheng S.-Y. Wei Z.-S. Li S. Zhang S.-J. Xie C.-F. Yao D.-S. (2020). Near-infrared reflectance spectroscopy-based fast versicolorin A detection in maize for early aflatoxin warning and safety sorting. *Food Chem.* 332:127419. 10.1016/j.foodchem.2020.127419 32622190

[B62] Zhu S. Zhang J. Chao M. Xu X. Song P. Zhang J. (2020). A rapid and highly efficient method for the identification of soybean seed varieties: Hyperspectral images combined with transfer learning. *Molecules* 25:152. 10.3390/molecules25010152 31905957 PMC6982693

